# Neanderthal and Denisovan retroviruses in modern humans

**DOI:** 10.1016/j.cub.2013.10.028

**Published:** 2013-11-18

**Authors:** Emanuele Marchi, Alex Kanapin, Matthew Byott, Gkikas Magiorkinis, Robert Belshaw

**Affiliations:** 1 Department of Zoology, University of Oxford, Oxford OX1 3PS, UK; 2 The Wellcome Trust Centre for Human Genetics, Oxford OX3 7BN, UK; 3 School of Biomedical and Healthcare Sciences, Plymouth University, Plymouth PL4 8AA, UK; 4 Virus Reference Department, Public Health England, London, UK

## Abstract

In the June 5th 2012 issue of *Current Biology*, Agoni *et al.*[1] reported finding 14 endogenous retrovirus (ERV) loci in the genome sequences of Neanderthal and/or Denisovan fossils (both ∼40,000 years old) that are not found in the human reference genome sequence. The authors [1] concluded that these retroviruses were infecting the germline of these archaic hominins at or subsequent to their divergence from modern humans (∼400,000 years ago). However, in our search for unfixed ERVs in the modern human population, we have found most of these loci. We explain this apparent contradiction using population genetic theory and suggest that it illustrates an important phenomenon for the study of transposable elements such as ERVs.

## Main Text

**Figure 1:**
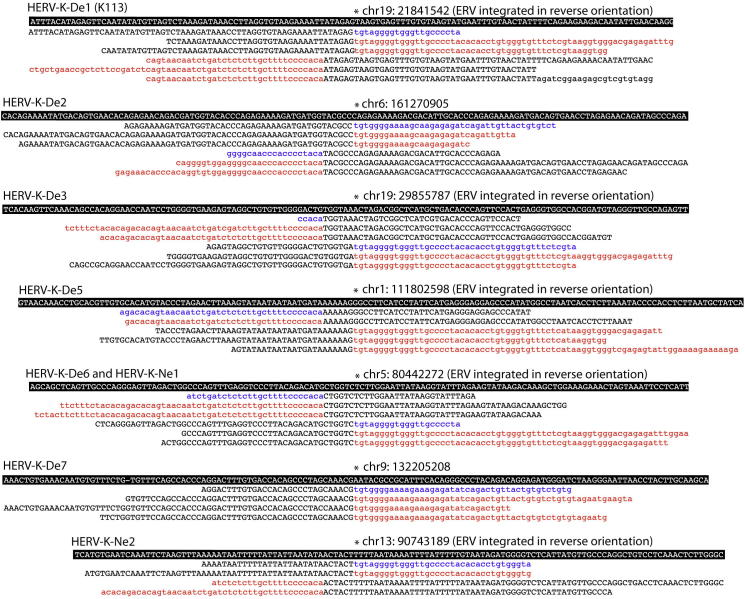
ERV loci absent from the human reference genome but present in both archaic hominins and modern humans. For each Agoni *et al*. [1] locus that we recovered in modern humans, the top sequence with black background shows the corresponding pre-integration region in the human reference sequence (hg19) and below are the reads from both the archaic hominins (with the viral regions in blue) and modern humans (viral regions in red). ‘De’ = Denisovan, ‘Ne’ = Neanderthal. In most cases there are reads spanning both upstream and downstream boundaries of the ERV, with the characteristic six base Target Site Duplication (TSD) of the host genome between them (see also Figure S1; only a small sample of the available reads from modern humans is shown). An asterisk shows the first base of the ERV, which in five of the seven instances represented here has integrated in reverse orientation. Coordinates taken from the UCSC Genome Browser at http://genome.ucsc.edu/ (Feb. 2009 (GRCh37/hg19) assembly). Both we and Agoni *et al*. [1] found the same A/G substitution in the TSD of HERV-K-De3.

The genomes of extinct human groups (archaic hominins), such as Neanderthals, are now available with high throughput sequencing technology, which can produce millions of short (∼100 base) sequences called reads from fossil bone or teeth. An analysis of a Neanderthal and a Denisovan genome identified many reads that contained sequences of viral origin, similar to known integrations of retroviruses into the germline of modern humans [1]. Such so-called endogenous retroviruses (or ERVs) are common, making up ∼5% of our genome. Some of the reads spanned the integration site of an ERV, called here a locus, and thus were part viral DNA and part archaic hominin DNA (Figure 1). In some cases, the authors [1] did not find an ERV at the corresponding coordinate in the human genome reference sequence. Instead they found the pre-integration site, which is the sequence that existed before the virus inserted a copy of itself into the chromosome. All of these loci belonged to one ERV lineage (family), called HERVK(HML2) or HERVK, which is the only lineage that has continued to replicate within humans in the last few million years [2]. They concluded that these retroviruses had infected the germline of the archaic hominins either after their divergence from modern humans (∼400,000 years ago) or immediately before divergence (with the integration and pre-integration sites then segregating differently in the lineages). However, while searching many new genome sequences of modern humans for ERVs, we have found most of these loci. For example, of the eight Denisovan loci for which Agoni *et al.* [1] were able to give precise genome coordinates, at least seven exist in modern humans. We have found six in an analysis of 67 cancer patient genomes (Figure 1), and examination of another study of 43 such genomes [3] shows all seven to be present (Supplemental information). One is K113 (19p12b), which is well-described and has a frequency of 16% in modern humans [2]. The four reported Denisovan loci lacking coordinates are within repetitive or unassembled regions of the genome, and we can neither confirm nor refute their presence in the modern human population: e.g. two loci are in transposable elements called Alu’s, of which there are ∼1,000,000 copies in the human genome (making up ∼10% of the human genome sequence). When an ERV integrates into another transposable element, finding this ERV locus can be a formidable computational challenge because there are many paralogous copies of the integration site. Two additional loci were reported from the Neanderthal fossil, and we have found one of these.

It is unlikely that these ERV loci in the archaic hominins are contaminants from modern human DNA. Average coverage of the Denisovan genome was only about twofold and the contamination rate among the reads was estimated using several approaches to have been less than 1% [4]. We believe that the explanation lies in fundamental population genetics. With the exception of co-opted ERV loci such as syncytins [5], which could increase in frequency due to positive selection, we assume ERV loci become common by genetic drift, and the average time for a neutral allele to go to fixation is 4N_e_ generations (where N_e_ is the effective population size). Given estimates of long-term human generation time and population size [6], this is ∼800,000 years. The population divergence of modern humans from the Denisovan/Neanderthal lineage is more recent, between 170,000 and 700,000 years according to a more recent — and much deeper —sequencing of the above Denisovan fossil [7], so many loci will have persisted at fluctuating frequencies in all three lineages.

As well as showing how differences in loci between one genome and another must be interpreted cautiously, our finding illustrates how single genomes, whether the human reference or one from an archaic hominin fossil, are likely to only contain those ERV loci that after almost a million years have drifted to high frequency. These old loci give us only a limited insight into the processes that created them, e.g. they will have accrued multiple inactivating mutations during this time. In contrast, loci that have integrated recently are more likely to produce proteins and might even be replicating. Such loci are interesting, perhaps most importantly because they are more likely to be pathogenic. The long-running debate over whether or not ERVs cause disease in humans has been handicapped by our poor knowledge of ERV polymorphism. Characterising individual loci is necessary to test ERV involvement in disease 8, 9, and will aid the potential exploitation of ERV proteins as cancer and HIV immunotherapy targets [10].

ERVs in fossil hominins also improve our understanding of both ERV and human evolution. When the ERV loci in modern humans have been reasonably well-sampled, fossil loci will help us build a robust mathematical model of ERV proliferation. Then, because ERV loci make easily detectable and irreversible genetic markers (the common mechanism called ‘recombinational deletion’ leaves a relict structure called a solo-LTR [9]), they might help us in the measurement of divergence dates and population sizes for these archaic hominins.

## Supplemental Information

Document S1. Experimental Procedures one table and one figure
