# Supplementary material for: Neanderthal and Denisovan retroviruses in modern humans
Source: Curr Biol. 2013 Nov 18;23(22):R994–5. doi: 10.1016/j.cub.2013.10.028 (PMC3923971; doi:10.1016/j.cub.2013.10.028)
Supplement: Document S1. Experimental Procedures one table and one figure [file mmc1.pdf]

Supplemental Information

Neanderthal and Denisovan retroviruses in modern humans

E Marchi, A Kanapin, M Byott, G Magiorkinis and R Belshaw

We found five of the seven loci also found in the archaic hominins (Figure 1 excluding De6/Ne1) among 21 Cancer Genome Atlas Project (TCGA) whole genome sequences and De6/Ne1 among 46 cancer patients in the WGS500 whole genome sequences (Oxford-Illumina consortium). In Table S1 we give details of the loci also recovered in another study. The only locus we have not also found, HERV-K-De4, was recorded only from one patient in this other study.

Supplemental Experimental Procedures

Cancer Genome Atlas Project (TCGA) whole genome sequences obtained with Illumina paired-end technology were downloaded as BAM files from the University of California, Santa Cruz's (UCSC) Cancer Genomics Hub (CGhub). ERV integrations that are absent from the human reference genome were detected in genomes using a combination of paired-end and chimeric read approaches as follows.

We first ran RetroSeq [S1], a program that uses paired-end Next (Second) Generation Sequencing (NGS) reads to detect new integration sites of a transposable element in a genome. It does this by finding in the BAM file those paired reads in which one read has been mapped to the human genome at a single location (henceforth called the anchor) and its paired read both (a) does not map nearby in the genome and (b) matches a reference transposable element. As a reference for the transposable element we used the recently integrated HERVK locus called K113 [S2]. A HERVK locus (provirus) consists of several genes flanked by two ~1000 base non-coding regions that are identical at the time of integration called LTRs (Long Terminal Repeats). We downloaded the LTR sequence of K113, whose two LTRs are identical reflecting its recent origin, from GenBank (accession

AY037928). We ran RetroSeq with default settings except for (a) requiring more stringency in the match to K113, namely 90% identity over at least 60 bases (reads were 100 bases), and (b) using the 'align' option 'exonerate' for a better, though more computationally intensive, alignment of the read to the K113 LTR.

We then used our own clustering algorithm to group anchors that might result from a novel HERVK locus. As part of this, we filtered out clusters that (a) resulted from the common unfixed loci belonging to the non-autonomous transposable element called SVA, which contains fragments of a HERVK LTR [S3], (b) were in regions with abnormally high coverage (and hence sometimes generated apparent anchors by chance), or (c) were in a genomic region close to where the reference sequence has a HERVK or similar locus. The last filtering is necessary as there are 'gray areas' in detecting matches, and possibly also sequence differences between homologous loci in the reference and TCGA genomes, which generated many spurious clusters. We therefore excluded reads mapping within 200 bases of one of the following RepeatMasker regions: HERVK, HERVK14C, HERVK3, HERVK9, LTR5, LTR5_Hs, LTR5A, LTR5B, and, as mentioned already, SVA.

This approach works well when the ERV integration is within a single copy region of the host genome. A major problem occurs when the ERV had integrated into another transposable element. In such cases the anchor might map equally well to many, potentially thousands, of positions around the genome. In practice, this leads to a dilution of the number of paired-end reads that show an ERV integration that is not in the genome reference sequence. These small clusters derived from novel ERV loci are then easily lost among the many other small clusters generated by the phenomena mentioned in the preceding paragraph (false positives).

Alongside the above analysis of paired-end reads, we therefore searched for chimeric single reads (i.e. reads which span the integration site) by selecting all reads that (a) did not map perfectly to the reference genome according to its CIGAR value and (b) had an eight base match to the start or end (sense and anti-sense) of

the HERVK LTR (determined using regex in perl). We then trimmed off any possible LTR sequence and then re-mapped the resulting trimmed reads to the genome sequence.

The coordinates of clusters found by RetroSeq after filtering were then matched with the coordinates of the re-mapped trimmed reads using the 'intersect' command in BEDTools [S4], and we confirmed novel integrations by finding chimeric reads within the resulting regions. We found chimeric reads by first selecting all reads that at least partially matched the region of the genome sequence spanning the above coordinates using the blastn option of BLAST [S5], and then aligning these reads using our own perl script (BreakAlign) to produce multiple alignments such as those shown in Figure 1. If a new ERV integration is present, the BreakAlign output will contain chimeric reads that span an ERV integration with (a) the part of the read that aligns to the genome sequence in upper case, and (b) the part of the read that is from the ERV in lower case. The output will contain chimeric reads spanning both ends of the integration (the beginning of the 5' LTR and the end of the 3' LTR), and these two ends will be separated in the multiple alignment by the typically six base Target Site Duplication (which results from the staggered cut made in the host double stranded DNA by the viral enzyme Integrase).

WGS500 whole genome sequences were searched by first finding unmapped reads with matches to the K113 LTR detected using BLAT [S6]. The matching regions were then removed and the trimmed reads re-mapped to the human genome reference. Putative chimeric reads that mapped within RepeatMasker [S7] entries were filtered out and the remaining reads analysed using BreakAlign as described above.

Table S1. Coordinates of archaic ERV loci found in Supplementary Material (Table S6) of Lee *et al.* [S8].

Agoni *et al*. name	chromosome	coordinate (hg19)	Lee *et al.* name
HERV-K-De1	19	21841542	ERVK_26
HERV-K-De2	6	161270905	ERVK_12
HERV-K-De3	19	29855787	ERVK_28
HERV-K-De4	11	60449865	ERVK_18
HERV-K-De5	1	111802598	ERVK_1
HERV-K-De6/Ne1	5	80442272	ERVK_10
HERV-K-De7	9	132205208	ERVK_16
HERV-K-Ne2	13	90743189	ERVK_22

Figure S1.

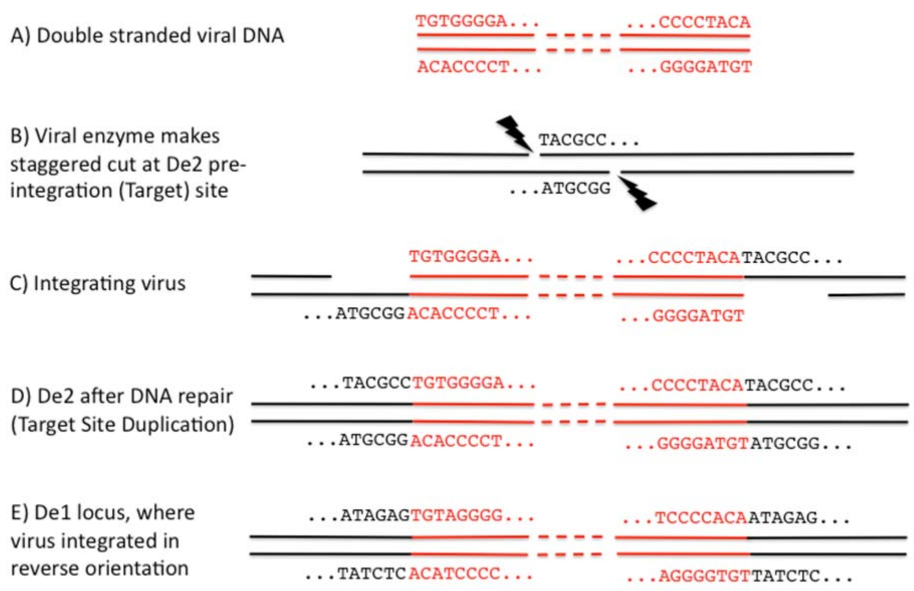

Figure S1. Diagram showing how ERV integration produces chimeric NGS reads such as those shown in Figure 1.

Panels A-D use as an example locus De2. After reverse transcription, viral double-stranded DNA (red) is integrated into the human chromosome (black). The viral integrase enzyme makes a staggered cut, typically of six bases, into which the viral DNA is inserted. DNA repair of the now single-stranded DNA on either side of the integration produces six identical bases (the TSD) flanking the virus. However, the virus might integrate in reverse orientation and in panel E locus De1 is shown as an example where this has occurred (note the changed viral sequence).

Supplemental References

S1. Keane, T.M., Wong, K., and Adams, D.J. (2012). RetroSeq: transposable element discovery from next-generation sequencing data. Bioinformatics *29*, 389-390.

S2. Turner, G., Barbulescu, M., Su, M., Jensen-Seaman, M.I., Kidd, K.K., and Lenz, J. (2001). Insertional polymorphisms of full-length endogenous retroviruses in humans. Curr. Biol. *11*, 1531-1535.

S3. Ostertag, E.M., Goodier, J.L., Zhang, Y., and Kazazian, H.H. (2003). SVA elements are nonautonomous retrotransposons that cause disease in humans. Am. J. Hum. Genet. *73*, 1444-1451.

S4. Quinlan, A.R., and Hall, I.M. (2010). BEDTools: a flexible suite of utilities for comparing genomic features. Bioinformatics *26*, 841-842.

S5. Altschul, S.F., Gish, W., Miller, W., Myers, E.W., and Lipman, D.J. (1990). Basic local alignment search tool. J. Mol. Biol. *215*, 403-410.

S6. Kent, W.J. (2002). BLAT – the BLAST-like alignment tool. Genome Res. *12*, 656-664.

S7. Smit, A.F.A., Hubley, R., and Green, P. (1996-2010). RepeatMasker Open-3.0. http://www.repeatmasker.org.

S8. Lee, E., Iskow, R., Yang, L., Gokcumen, O., Haseley, P., Luquette III, L.J., Lohr, J.G., Harris, C.C., Ding, L., Wilson, R.K., et al. (2012). Landscape of somatic retrotransposition in human cancers. Science *337*, 967-971.
